# The Aging Substantia Nigra is Characterized by ROS Accumulation Potentially Resulting in Increased Neuroinflammation and Cytoskeletal Remodeling

**DOI:** 10.1002/adbi.202400814

**Published:** 2025-03-12

**Authors:** Britta Eggers, Simone Steinbach, Isabel Gil Aldea, Sharon Keers, Mariana Molina, Lea T. Grinberg, Helmut Heinsen, Renata E. Paraizo Leite, Johannes Attems, Caroline May, Katrin Marcus

**Affiliations:** ^1^ Medizinisches Proteom‐Center Medical Faculty Ruhr‐University Bochum 44801 Bochum Germany; ^2^ Medical Proteome Analysis Center for Protein Diagnostics (PRODI) Ruhr‐University Bochum 44801 Bochum Germany; ^3^ Navarrabiomed Biobank Hospital Universitario de Navarra Pamplona Navarra 31008 Spain; ^4^ Institute of Neuroscience and Newcastle University Institute for Ageing Newcastle University Newcastle upon Tyne NE1 7RU UK; ^5^ Physiopathology in Aging Lab/Brazilian Aging Brain Study Group‐LIM22 University of São Paulo Medical School São Paulo CEP 01246 903 Brazil; ^6^ Institute of Forensic Medicine University of Wuerzburg Versbacher Str. 3 97078 Wuerzburg Germany; ^7^ Department of Neurology Memory and Aging Center University of California San Francisco CA 94158 USA; ^8^ Discipline of Geriatrics University of São Paulo Medical School São Paulo NE1 7RU Brazil; ^9^ Translational and Clinical Research Institute Newcastle University Edwardson building, Campus for Ageing and Vitality Newcastle‐upon‐Tyne NE4 5PL UK

**Keywords:** aging, data independent acquisition, dopaminergic neurons, laser microdissection, proteomics, substantia nigra

## Abstract

Aging is a progressive and irreversible process, serving as the primary risk factor for neurodegenerative disorders. This study aims to identify the molecular mechanisms underlying physiological aging within the *substantia nigra*, which is primarily affected by Parkinson's disease, and to draw potential conclusions on the earliest events leading to neurodegeneration in this specific brain region. The characterization of essential stages in aging progress can enhance knowledge of the mechanisms that promote the development of Parkinson's disease. To gain a comprehensive overview three study groups are utilized: young individuals (mean age: 28.7 years), middle‐aged (mean age: 62.3 years), and elderly individuals (mean age: 83.9 years). Using the proteomic approach, crucial features of physiological aging are able to be identified. These include heightened oxidative stress, enhanced lysosomal degradation, autophagy, remodeling of the cytoskeleton, changes in the structure of the mitochondria, alterations in vesicle transportation, and synaptic plasticity.

## Graphical abstract

Definition of study groups, including young individuals (*n* = 7 mean age: 28.7 years), middle aged individuals (*n* = 14 mean age: 62.3 years) and elderly individuals (*n* = 14 mean age: 83.9 years). Condensed workflow including sample preparation (sample lysis and tryptic digestion), mass spectrometric measurements and subsequent data and statistical analysis. The proteomic changes that occur during the aging process are highlighted, whereby alterations in vesicle transport, mitochondria, lysosomal degradation and autophagy, synaptic plasticity and increased oxidative stress was observed. The figure was created using BioRender.com.

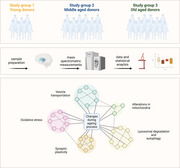

## 1 Introduction

Aging is a highly complex process and one of the major risk factors for developing a variety of diseases. Hence, numerous research groups and studies focus on elucidating age‐related changes of the human body.^[^ ^1^, ^2^, ^3^ ^]^ Especially the aging brain lies in the focus of research, as the risk of developing a neurodegenerative disease is increasing exponentially.^[^ ^4^ ^]^ Furthermore, the brain is particularly sensitive to the aging process, leading to structural changes in brain morphology and size, vasculature, and neural interactions causing cognition and increasing the risk of stroke and ischemia.^[^ ^5^, ^6^, ^7^ ^]^ On the molecular level, physiological aging is often accompanied by increased mitophagy, cellular senescence, and an increase in oxidative stress eventually leading to neuroinflammation.^[^ ^8^, ^9^ ^]^

Morphological alterations aberrant in the aging brain of healthy individuals are also found and defined as classical hallmarks of neurodegeneration, such as neuronal loss, gliosis as well as loss of myelination.^[^ ^10^ ^]^ On the molecular level physiological aging and neurodegeneration are both associated with disrupted calcium homeostasis, enhanced neuroinflammation, and oxidative stress.^[^ ^11^, ^12^ ^]^ Furthermore, both physiological aging and neurodegeneration exhibit mitochondrial alterations, protein aggregation, and a disrupted protein clearance system.^[^ ^13^ ^]^ Neurodegenerative diseases frequently exhibit distinctive pathologies due to the selective vulnerability of certain brain regions and cell types. This raises the question of whether the changes observed in physiological aging can predict the earliest signs or risk factors of neurodegeneration.

Therefore, it is crucial to identify region‐specific changes in physiological brain aging. This provides insight into the mechanisms that may influence the development of neurodegenerative diseases. However, human brain samples are a limited resource, and comprehensive analysis is often not possible due to the unavailability of all brain areas. Thus, the majority of studies utilize model organisms, to study age‐related changes in the substantia nigra (SN).^[^ ^14^, ^15^, ^16^ ^]^ But, unlike humans, most model organisms lack the pigmentation, caused by neuromelanin deposits, within dopaminergic neurons of the SN. Loss of pigmentation is a major hallmark of synucleinopathies,^[^ ^17^, ^18^ ^]^ highlighting its significance in disease progression.

To date, only a limited number of studies have investigated age‐related changes in the human SN. A 3D structural study verified a smaller SN volume in aged individuals, although the number of neurons remained stable.^[^ ^19^ ^]^ Histological studies have shown moderate changes in the population of astrocytes and microglia.^[^ ^20^, ^21^ ^]^ An increase in iron load with age^[^ ^22^ ^]^ and deficiencies in the respiratory chain, including high levels of mtDNA deletions, in aged dopaminergic neurons were also detected.^[^ ^23^ ^]^ Another study focusing on age‐related changes in the human SN on the molecular level, identified significant oxidation and nitration of proteins, as well as a decrease in essential antioxidant molecules and enzymes such as GSH, SOD, catalases, GPx, and GR, compared to other brain regions.^[^ ^24^ ^]^ However, to our knowledge, no global proteomic‐based studies of the human SN in the context of aging have been performed so far.

Our established and recently revised workflow combines laser microdissection and mass spectrometry to utilize minimal amounts of tissue and further isolate specific substructures for the precise analysis of local proteomic changes within the human brain.^[^ ^25^, ^26^ ^]^ With our approach, we already successfully characterized the proteome of the SN in healthy individuals and Dementia with Lewy bodies (DLB) patients,^[^ ^27^ ^]^ whereby ongoing oxidative damage and impairment of protein degradation could be observed in the patient study group. However, early pathologic changes remain unclear, due to the fact that post‐mortem brain tissue samples of patients, often represent the latest disease stages. Therefore, understanding the impact of healthy aging on the molecular level of the SN, may lead to insights into the earliest events causing symptoms of synucleinopathy. This study aims to characterize the effect of aging on the SN by determining protein profiles in young (*n* = 7), middle‐aged (*n* = 14), and elderly (*n* = 14) individuals at the protein level.

## 2 Result

**Figure 1:**
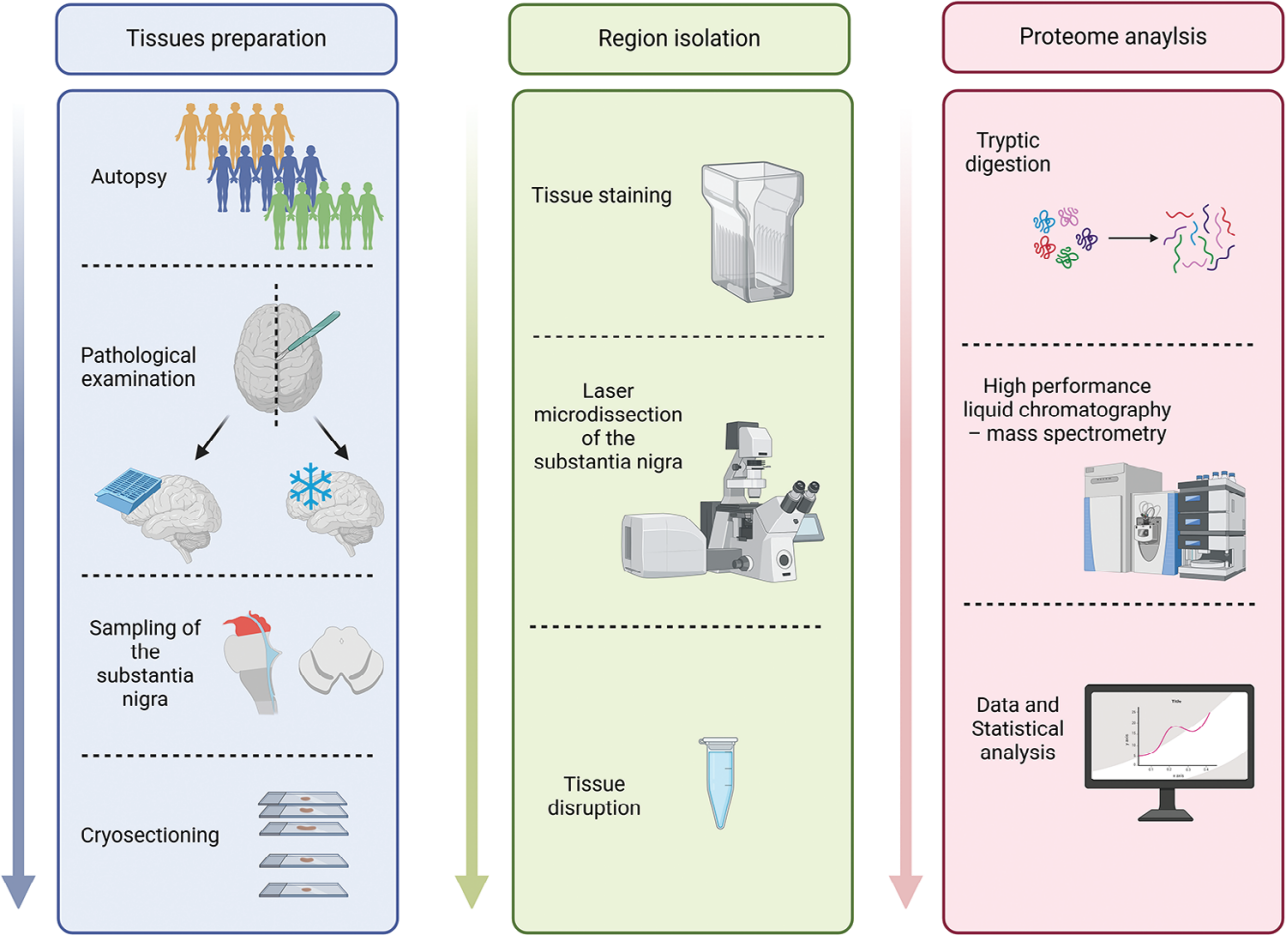
Graphical Workflow. Donated post‐mortem brains are pathologically examined, the *substantia nigra* isolated and cryosected in 10 µm slices onto laser microdissection‐compatible PEN‐slides. The slides are stained utilizing cresyl violet staining to visualize neurons and areas of interest are excised. The resulting sample is lysed and tryptically digested into peptides prior to mass spectrometric measurements. The resulting raw data is analyzed and statistically evaluated. Created in BioRender. May, C. (2025) https://BioRender.com/m18z266.

The aging study involved 35 SN samples from 35 donors. SN tissue was excised for each donor specifically by laser microdissection and subsequent mass spectrometric measurements were carried out to address the question of physiological aging‐related alterations within this brain area. An overview of the workflow is given in **Figure** **1**.

### 2.1 Aging Contributes to Shaping the Protein Pattern Present, but with Only Mild Effects on the Total Proteome

**Figure 2:**
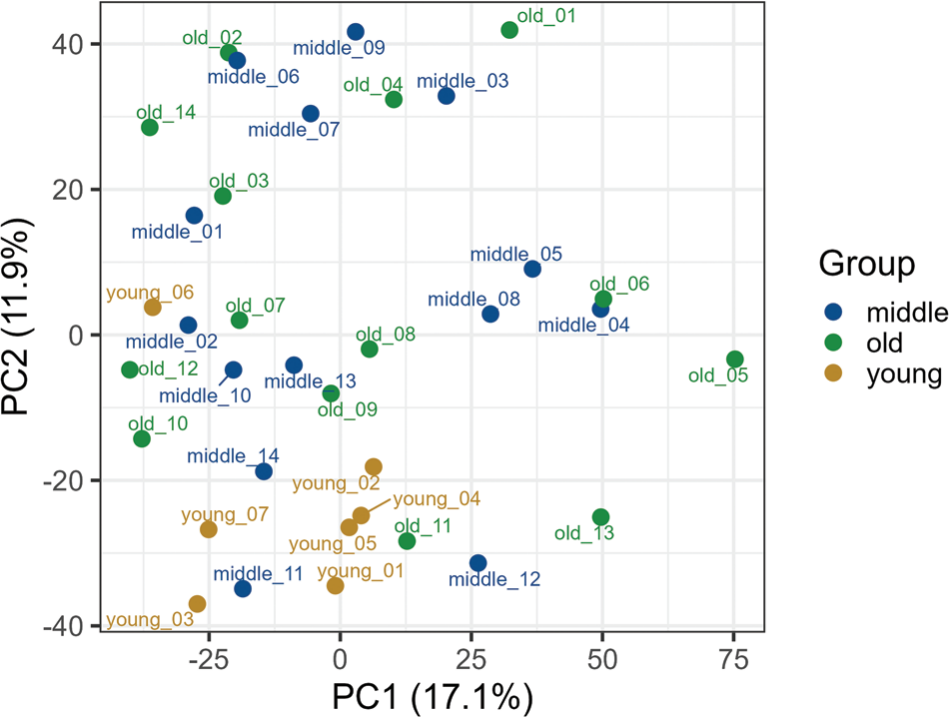
Principal component analysis of young (yellow), middle‐aged (blue), and old‐aged (green) SN tissue. Young individuals (yellow) discretely cluster, however no distinct separation of the two other age groups could be observed.

Proteomic profiling identified 3806 proteins. Our initial aim was to investigate the effect of aging on the proteome of the SN through sample clustering analysis. The results of the principal component analysis (PCA, see **Figure** **2**) showed mild clustering of samples from young individuals (in yellow). In contrast, samples from middle‐aged individuals (in blue) and elderly individuals (in green) did not form distinct groups. Additionally, we aimed to determine whether sex may influence clustering in the PCA. For the four included female subjects a mild subcluster was observable for the old study group (see Figure 2, old_01, old_02, old_14) however also male subjects of the same age range were found in near proximity (old_03, old_04), hence a sex‐specific bias in our dataset should not be expected. For the middle‐aged cohort the female subject (middle_05), clustered together with two other male subjects of the same age range, as well as two male subjects of the old study group. In summary, we concluded, that aging contributes to shaping the protein pattern present but only has mild effects on the total proteome.

### 2.2 Aging Changes the Abundance of Different Cell Types Present in the SN

**Figure 3:**
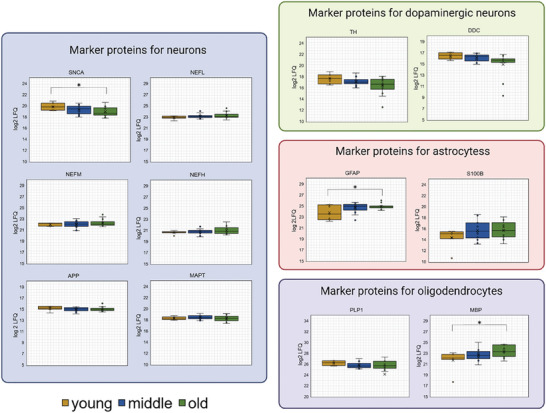
Changes of Cell marker proteins during aging. The box plots show the abundance of known cell‐type markers based on LFQ values. Neurons are shown in blue, dopaminergic neurons in green, astrocytes in red, and oligodendrocytes in purple. The boxplots are color‐coded to represent young individuals in yellow, middle‐aged individuals in blue, and elderly individuals in green. Any significantly differentially expressed proteins (*p*‐value <0.05) are marked with an asterisk. Figure created with Biorender.com.

To investigate whether aging affects the abundance of various cell types within the SN tissue, we examined the levels of established cell type‐specific markers, including neurons, dopaminergic neurons, astrocytes, and oligodendrocytes (refer to **Figure** **3** and Table S4, Supporting Information “cell types”). Out of the six general neuronal markers, NEFL, NEFM, NEFH, APP, and MAPT did not show any significant changes in abundance across different age groups. However, SNCA was found to be enriched by 1.8‐fold in young individuals compared to the old study group (student's *t* test *p*‐value <0.05). Markers for dopaminergic neurons (TH, DDC) showed a slightly increased abundance in the young study group compared to both middle‐aged and old subjects. However, the observed increase did not reach statistical significance. The astrocytic marker GFAP exhibited high variability in young subjects and a significant 2.17‐fold decrease in the eldest subjects compared to the young group (student's *t* test *p*‐value <0.05). Furthermore, the abundance of the second astrocytic marker, S100B, varied greatly between middle‐aged and old individuals, showing a slight enrichment but no statistical significance. The oligodendrocytic marker MBP displayed a significant 3.3‐fold increase in the old study group compared to young individuals (student's *t* test *p*‐value <0.05). However, no significant increase was observed for PLP1.

### 2.3 The Aging SN is Characterized by Increased Oxidative Stress, Enhanced Neuroinflammation, and an Increase in the Immune Response

As a next step, we aimed to globally assess proteins that significantly change in abundance in response to aging. To achieve this, we performed an analysis of variance (ANOVA) to identify proteins that showed significant changes across all three comparisons (one‐way ANOVA *p*‐value < 0.05, PostHoc Tukey's HSD test for one‐way ANOVA FDR 0.05, see Table S4, Supporting Information ANOVA). Our findings revealed proteins displayed increasing (see **Table** **1**) or decreasing (see **Table** **2**) abundance during the aging process. To visualize the effects of physiological aging, we calculated the enrichment (fold change (FC)) using log2 label‐free quantified values (LFQ) between middle‐aged and young individuals or old and young individuals. To determine whether the observed proteins underwent gradual enrichment or depletion throughout the aging process, the FC difference was computed. FC differences greater than 1 were considered as continuous enrichment with age, those greater than 0.5 as slight gradual enrichment, and those less than 0.5 as marginal enrichment. For a comprehensive understanding, all differential proteins were annotated based on their function or location and sorted accordingly. Out of the 59 proteins that increase during the aging process, only a few, namely CP, CAPS, and ICAM1, showed a fold change difference over 1, indicating a continuous enrichment throughout the aging process. Four proteins, namely C4A, TPP1, CLBYL, and FLNC, displayed a mild increase over the aging process with a fold change difference greater than 0.5. The proteins that remained had either already reached their maximum enrichment in the middle‐aged group or showed only minimal differences in fold change between young and elderly individuals. Functionally, the proteins that were consistently affected by aging were found to be associated with inflammatory processes, immune response, autophagy, and oxidative stress (see Table 1).

**Table 1:** Proteins were found to be of higher abundance in middle‐aged and elderly individuals compared to young individuals sorted after functional categories (one‐way ANOVA *p*‐value <0.05 and subsequent PostHoc Tukey's HSD test for one‐way ANOVA FDR 0.05) Gene symbols represent the differential proteins, which have been annotated based on their function or localization. The fold changes (FC) were calculated using log2 LFQ values and are displayed for both comparisons (middle/young; old/young). To determine whether the observed proteins underwent gradual enrichment or depletion throughout the aging process, the FC difference was computed. FC differences greater than 1 were considered as continuous enrichment with age, those greater than 0.5 as slight gradual enrichment, and those less than 0.5 as marginal enrichment.

Category	Protein (Gene name)	Function/Localization	FC middle/young	FC old/young	FC difference
Immune reaction and Inflammation					
	CP	inflammation	2.40	4.08	1.68
	C4A	inflammation	2.14	2.95	0.81
	HLA‐DRB1	inflammation	2.57	3.05	0.49
	LGALS3	inflammation	2.05	2.49	0.44
	CAPG	immune reaction	1.74	2.16	0.43
	LYZ	immune reaction	3.12	3.30	0.19
	LPCAT2	inflammation	1.59	1.73	0.13
	CD44	inflammation	3.07	3.19	0.12
	C1QC	inflammation	2.00	2.10	0.10
	DPP3	angiotensin system	1.36	1.45	0.09
	GGT5	immune reaction	1.60	1.68	0.08
	TGM2	angiogenesis	1.68	1.72	0.04
	ANXA3	angiogenesis	1.49	1.53	0.04
Degradation, oxidative stress, apoptosis					
	TPP1	lysosome	2.85	3.62	0.77
	RNF13	apopotosis	2.04	2.53	0.49
	SOD3	oxidative stress	1.93	2.37	0.45
	DNASE2	apopotosis	2.49	2.75	0.26
	CTSZ	lysosome	1.68	1.85	0.16
	SCARB2	lysosome	1.46	1.59	0.13
	PRCP	lysosome	1.41	1.50	0.10
	HINT1	proteasomal degradation	1.86	1.94	0.08
	PRDX6	oxidative stress	1.49	1.50	0.01
Neuronal maintenance and metabolism					
	CAPS	cell signaling	5.63	7.03	1.40
	ICAM1	adhesion	3.89	4.92	1.03
	CLYBL	B12 metabolism	1.59	2.18	0.59
	FLNC	cytoskeleton	1.98	2.53	0.55
	SLC14A1	urea transport	2.47	2.95	0.48
	PLP2	cell differentiation	2.19	2.54	0.35
	HOGA1	metabolism	1.59	1.83	0.24
	MYLK	smooth muscle contraction	1.66	1.89	0.23
	GALM	metabolism	1.84	2.03	0.20
	ACSS3	metabolism	1.40	1.59	0.19
	RPE	pentose phosphate pathway	1.96	2.15	0.19
	CAVIN2	caveolar biogenesis	1.93	2.11	0.17
	COL6A3	cytoskeleton	2.01	2.16	0.16
	STARD10	metabolism	1.49	1.62	0.13
	APRT	metabolism	1.80	1.91	0.12
	NAMPT	metabolism	1.42	1.53	0.11
	APOD	lipoprotein metabolism	2.17	2.26	0.10
	MSN	cytoskeleton	1.37	1.46	0.10
	AZGP1	lipid degradation	2.16	2.24	0.08
	AK2	hematopoiesis	1.47	1.53	0.06
	SRSF7	metabolism	1.53	1.57	0.04
	PAICS	metabolism	1.24	1.27	0.03
	SUCLG2	metabolism	1.62	1.64	0.02
	LMNB1	filaments	1.52	1.53	0.02
	CLU	chaperone	1.42	1.43	0.01
	CLU	chaperone	1.42	1.43	0.01
	RDX	cytoskeleton	1.40	1.40	0.01
DNA Replication and Transcription					
	CAVIN1	transcription	1.54	1.84	0.30
	PLCD1	mRNA splicing	1.45	1.56	0.11
	FUBP3	gene expression	1.39	1.48	0.09
	PRPSAP2	DNA metabolism	1.30	1.37	0.07
	DUT	DNA replication	1.35	1.41	0.06
	EDF1	transcription	1.81	1.85	0.04
	MACROH2A1	transcription	1.27	1.30	0.03
Vesicles					
	SNAP23	vesicle trafficking	1.51	1.83	0.31
	SCIN	exocytosis	1.68	1.74	0.06
Others					
	CPNE3	membrane‐binding proteins	1.26	1.34	0.08

**Table 2:** Proteins were found to be of lower abundance in middle‐aged and elderly individuals compared to young individuals sorted after functional categories (one‐way ANOVA *p*‐value < 0.05, and subsequent PostHoc Tukey's HSD test for one‐way ANOVA FDR 0.05). Gene symbols represent the differential proteins and are annotated based on their function or localization. The fold changes (FC) were calculated using LFQ values and are displayed for both comparisons (young/middle; young/old). To determine whether the observed proteins underwent gradual enrichment or depletion throughout the aging process, the FC difference was computed. FC differences greater than 1 were considered as continuous enrichment with age, those greater than 0.5 as slight gradual enrichment, and those less than 0.5 as marginal enrichment.

Category	Protein (Gene name)	Function/Localization	FC young/middle	FC young/old	FC Difference
Vesicles, ER, and Golgi apparatus					
	DYNC1LI1	cargo transport	1.27	1.43	0.16
	EIPR1	vesicle transport	1.43	1.58	0.15
	WDR44	vesicle recycling	1.38	1.50	0.12
	RAP1GAP	RAP1 activator	1.38	1.50	0.12
	SNAP91	vesicle transport	1.27	1.38	0.11
	ACBD3	Golgi structure	1.55	1.65	0.10
	PDZD8	mitochondria/ER tethering	1.43	1.52	0.09
	AP3M1	vesicle trafficking	1.62	1.71	0.08
	ARFIP2	vesicle biogenesis	1.53	1.59	0.06
	UBQLN2	UPS system, ERAD	1.29	1.34	0.05
	VPS45	vesicle trafficking	1.17	1.22	0.05
	MRPL13	ribosome	1.66	1.71	0.05
	ABCB6	melanogenesis	1.57	1.60	0.03
	DNAJC10	ER folding, ERAD	1.58	1.61	0.03
	SYNJ1	endocytosis	1.26	1.29	0.03
	ATL1	membrane tethering	1.37	1.39	0.02
Neuronal maintenance					
	KCNQ2	neuronal excitability	1.65	2.01	0.37
	LINGO1	neg regulation myelination	1.64	1.93	0.30
	PRMT1	neurite outgrowth	1.56	1.72	0.16
	DBN1	dendrite morphology	1.65	1.76	0.11
	CLPTM1	GABAergic transmission	1.47	1.54	0.07
	YWHAZ	signal transduction	1.45	1.51	0.06
	ACHE	neurotransmitter hydrolysis	1.43	1.48	0.05
	RAB3GAP2	exocytosis of neurotransmitters	1.31	1.35	0.04
	TNR	neural extracellular matrix	1.21	1.24	0.03
	ARMCX3	mitochondria transport in axons	1.36	1.38	0.02
	MAP6	dendrite morphogenesis	1.38	1.40	0.01
	YWHAH	signal transduction	1.37	1.38	0.01
	STXBP5	neurotransmitter release	1.44	1.45	0.00
Lipids and Metabolites					
	HMGCS1	cholesterol metabolism	2.00	2.56	0.56
	DGKZ	lipid metabolism	1.44	1.67	0.23
	APOE	lipid transport	1.51	1.70	0.19
	OSBP	lipid transport	1.22	1.32	0.10
Others					
	WDR17	function not known	1.95	2.24	0.29
	YARS1	aminoaclyation of tRNA	1.27	1.36	0.10
	AKAP2	enzyme positioning	1.89	1.90	0.01

To further substantiate our findings, we searched for proteins associated with the above‐mentioned processes by directly comparing young and oldest individuals, since these two groups display the greatest age difference and therefore age‐related effects should be the strongest. The comparison led to the identification of 569 differential proteins, of whose 297 were found to be increased in the old study group (Student's *t*‐test *p*‐value < 0.05, see Table 1 and Table S4 (Supporting Information), “young versus old *t*‐test”). Among these, we identified 12 proteins associated with neuroinflammation, including proteins of the complement system and immune response and 14 proteins associated with the blood coagulation system. We also found eight lysosomal proteins and 19 proteins associated with apoptotic processes, as well as oxidative stress to be enriched, among them DNAJB2, a protein essential for the functional recovery of PRKN. As ROS is primarily produced through electron transfer within the respiratory chain, we checked our data for proteins associated with the five complexes. We identified five proteins associated with complex I, one protein of complex II, and CYCS to be increased in elderly individuals. Additionally, we noticed an increase in several calpain isoforms, which are all involved in proteolysis essential for cytoskeleton remodeling. We also found 46 additional proteins associated with cytoskeleton structure, stability, maintenance, and cell adhesion (for discussed proteins see Table S4, Supporting Information “increased old annotated”). Our in‐depth analysis leads us to conclude that the aging SN is characterized by increased oxidative stress, enhanced neuroinflammation, and a heightened immune response. Proteins involved in cytoskeletal remodeling were found to be increased. These processes may potentially be an important factor leading to elevated levels of proteins essential for the proper clearance of biological molecules and repurposing of metabolites.

In parallel, we assessed proteins that decreased during aging (one‐way ANOVA *p*‐value < 0.05, posthoc Tukey's HSD test for one‐way ANOVA FDR 0.05, see Table 2 and Table S4, Supporting Information, ANOVA). Unlike proteins that increase over time, those that decrease did not exhibit fold change differences greater than 1, indicating no continuous decline. Of the 36 identified proteins, the majority were associated with neuronal function, including maintenance, excitability, and transmission, as well as neurotransmitter release. Additionally, we identified a large number of proteins essential for vesicle trafficking and recycling. Interestingly, proteins necessary for the proper degradation of endoplasmic reticulum‐associated proteins (ERAD) by the proteasome system were found to be downregulated in elderly individuals. Two proteins that activate tyrosine 3‐monooxygenase (YWHAZ, YWHAH), an essential enzyme in the dopamine synthesis pathway, were found to decrease over the aging process. This may indicate alterations in dopamine synthesis.

**Figure 4:**
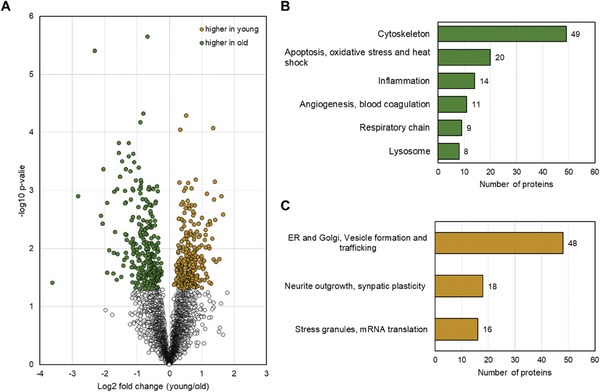
A) Differential Proteins. Volcano Plot displaying differential proteins (*p*‐value <0.05), being of higher (green) and lower (yellow) abundance in elderly subjects. B) Function and localization of increased proteins in the aged study group. Manual annotation of protein function and localization of proteins was found to be increased in aged individuals, as well as information on the number of proteins associated with said terms. C) Function and localization of decreased proteins in the aged study group. Manual annotation of protein function and localization of proteins were found to be decreased in aged individuals, as well as information on the number of proteins associated with said terms.

Our aim was to confirm our initial findings by analyzing proteins that were differentially expressed in a direct comparison between young and elderly individuals (Student's *t*‐test *p*‐value < 0.05, see **Figure** **4** and Table S4, Supporting Information “young versus old *t* test”). In total, 272 were found to have a decreased abundance in the old study group. Of those, we identified 18 proteins associated with neurite outgrowth and synaptic plasticity and 48 proteins associated with the ER, Golgi apparatus, and vesicle formation/trafficking. Notably, HABP4, one of the proteins with the highest fold changes, functions as a ribosome‐binding protein that promotes ribosome hibernation and prevents proteasomal degradation. In older subjects, certain proteins that regulate autophagy (RETREG, TRIM32), as well as those responsible for maintaining the proper distribution and morphology of mitochondria (OPA1, DNM1L, LARS1, ARMCX3), lysosomal homeostasis, and correct positioning of lysosomes (CLCN7, FAM241B, SPAG9, PIP4P1), were found to be decreased. Additionally, elderly individuals exhibited a decreased abundance of several proteasomal subunits (PSMB5, PSMC1/2, PSMD1/3/4). Surprisingly, we identified a vast number of proteins related to RNA processes that were of lower abundance in our old study group. These proteins include those that are essential for the formation of stress granules, RNA transport, and mRNA translation (for discussed proteins, see Table S4, Supporting Information “decreased old annotated”).

Our results suggest that the aging SN is characterized by changes in the organization of the mitochondria, ER, and Golgi apparatus. These changes can lead to difficulties in vesicle trafficking, which in turn affects proteins that are essential for synaptic plasticity and neurite outgrowth.

### 2.4 Young Individuals Display Higher Levels of Proteins Associated with Parkinson's Disease

**Figure 5:**
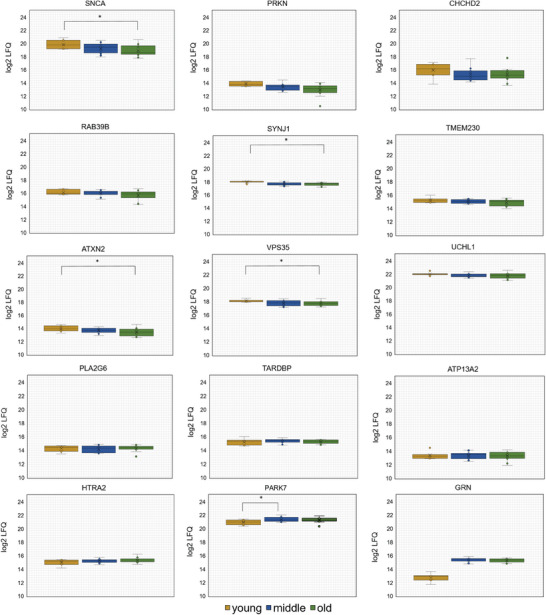
Boxplots displaying the abundance levels (based on LFQ) values of known Parkinson's disease‐associated proteins (identified by gene symbol). The boxplots illustrate the values of young, middle‐aged, and elderly individuals, marked in yellow, blue, and green, respectively. Any proteins that are significantly differentially expressed (*p*‐value < 0.05, student's *t*‐test) are marked with an asterisk.

As a final step, we wanted to assess how known PD‐associated proteins might be affected by the aging process (see **Figure** **5** and Table S4, Supporting Information “PD‐associated genes”). Our analysis revealed a decrease in the levels of certain proteins, including SNCA, PRKN, CHCHD2, RAB39B, SYNJ1, TMEM230, ATXN2, and VPS35, over the course of aging. Proteins with similar expression values among all age stages (UCHL1, PLA2G6, TARDB, ATP13A2, and HTRA2) were identified. Additionally, proteins increasing during aging (PARK7 and GRN) were found. Among these proteins, SNCA, SYNJ1, ATXN2, VPS35, and PARK7 were significantly different (student's *t* test *p*‐value < 0.05).

### 2.5 The Aging Brain Proteome—A Literature Comparison

To contextualize and validate the proteomic‐derived results of the aging substantia nigra a review of the literature was conducted. Unfortunately, proteomic analyses of human postmortem brain tissue in the context of aging are scarce. For this reason, we decided that studies utilizing other human‐derived tissues or body fluids, or proteomic studies conducted on rodent brains, should be included.

A recent systematic review and analysis of human proteomic aging studies identified key processes that change in response to age in various tissues and body fluids.^[^ ^28^ ^]^ Of the 36 studies assessed, a total of 32 proteins were identified to be commonly associated with aging (identified in at least five studies). Of these 32 proteins, 6 proteins were also identified in our study, including 3 proteins associated with inflammation (HP, C4A, FGA) and 3 proteins associated with the cytoskeleton (FN1, CAPN2, LAMC1). Enrichment studies conducted on all proteins identified to be altered in aging (1128 proteins, identified in at least two studies) revealed a substantial enrichment of terms such as complement and coagulation cascade‐associated, angiogenesis, immune activation, and extracellular organization, thereby substantiating the results of our study.

Moreover, we incorporated two additional aging studies that examined proteomic age‐related changes in the cortex, hippocampus, and brain endothelium of aging mice.^[^ ^29^, ^30^ ^]^ Again, we performed a comparative analysis of proteins that demonstrated increased abundance in the old cohort (see Figure S1, Supporting Information). Interestingly 3 proteins were found to be of higher abundance in all 4 data sets: MBP, GFAP, and LAMC1. Furthermore, a total of 29 additional proteins were identified as being consistent with the differential proteins identified in the present study across a minimum of one of the three data sets. Intriguingly, the most significant overlap was observed with mouse brain endothelium tissue, where 13 shared proteins were identified. It is noteworthy that overlapping proteins were mainly associated with the cytoskeleton, inflammation or oxidative stress, and autophagy.

## 3 Discussion

**Figure 6:**
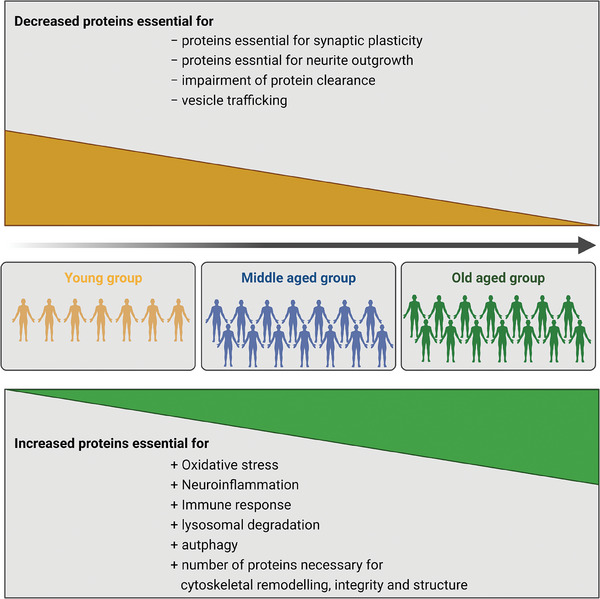
Overview of study results. Proteomics alterations within the substantia nigra (SN) are associated with the aging process. Aging is accompanied by an increase in oxidative stress, neuroinflammation, immune response, lysosomal degradation, and autophagy as well as cytoskeletal remodeling. Processes found to be decreased in the aged SN were associated with synaptic plasticity, neurite outgrowth, and vesicle trafficking. Created in BioRender. May, C. (2025) https://BioRender.com/i97r068.

The aim of this study was to identify the molecular mechanisms that contribute to physiological aging. Our analysis focused on the SN, with the goal of drawing conclusions about the earliest events that lead to neurodegeneration in this particular brain area. The SN is located in the midbrain and contains the highest density of neuromelanin granule‐containing dopaminergic neurons that project into the striatum, controlling physical movement. Pathological alterations in the brain, specifically the loss of neurons, are key hallmarks of Parkinson's disease (PD) and Dementia with Lewy Bodies (DLB). Identifying key events in the aging process can contribute to a better understanding of mechanisms that may exacerbate disease onset or progression. With our proteomic approach, we were able to confirm key aspects of physiological aging (for an overview see **Figure** **6**):

1. Elderly individuals exhibit an increase in oxidative stress.
2. Elderly individuals show increased neuroinflammation and a higher immune response.
3. Mechanisms essential for protein clearance and repurposing of metabolites, such as lysosomal degradation and autophagy, are increased.
4. Elderly individuals have increased levels of proteins required for the cytoskeletal structure, integrity, and remodeling.
5. Elderly individuals display alterations in the organization of the mitochondria, ER, and Golgi apparatus, leading to difficulties in vesicle trafficking.
6. Proteins necessary for synaptic plasticity and neurite outgrowth are reduced in elderly individuals.

The key aspects will be discussed in more detail in the following sections. All proteins to be discussed later were significantly enriched either by one‐way ANOVA (*p*‐value < 0.05 and subsequent PostHoc Tukey's HSD test for one‐way ANOVA FDR 0.05), or student's *t*‐test (*p*‐value < 0.05) unless otherwise stated.

However, we also must state, that our presented data and results have several limitations. First, we did not differentiate between cell types in our analysis, which will be a logical next step, since our results pointed toward an alteration in dopaminergic neuron abundance, as well as increased inflammation, a potential result of microglial activation. Further, we aimed to include all samples present in our institute in the study, to overcome the general high biological variability of tissue samples, resulting in an unequal number of male and female subjects. We are aware, that sex‐specific differences are an important biological factor. Nevertheless, since the number of females in our study was comparable small, we were unable to elaborate on potential sex‐specific differences. Still, we elucidated, whether a sex‐specific bias may be present in our data, as described in the result section. We further must note, that all results presented and discussed here are based on mass spectrometry‐derived data, only. The hypotheses and conclusions stated here should be verified in an experimental setting, e.g. by utilizing in vivo models of aging. However, all key aspects noted above, have been frequently described to be prevalent in brain aging, as outlined in our discussion below, substantiating our findings.

### 3.1 Elderly Individuals Exhibit Increased Oxidative Stress, Which May Result From Disturbed Mitochondrial Homeostasis and Dopamine Metabolism, as well as an Increase in Autophagy and Neuroinflammation

Currently, the “free radical theory of aging”^[^ ^31^ ^]^is widely accepted as the most plausible explanation for the mechanisms of basic aging. Mitochondria, specifically complex I and III, are the primary intracellular source of ROS during healthy aging.^[^ ^32^ ^]^ To counterbalance ROS production under physiological conditions, detoxification is carried out by superoxide dismutases, glutathione peroxidases, and periredoxins.^[^ ^33^ ^]^ To investigate the impact of mitochondria on ROS production, we quantified mitochondrial proteins. Our study identified 80% of proteins associated with complex I, 75% of complex II, 64% of complex III, 53% of complex IV, and all proteins forming complex V, the ATP synthase (see Table S4, Supporting Information “Respiratory Chain”). However, only a fraction of these proteins were found to be differentially expressed during the aging process. In our ANOVA‐based analysis, only AK2, which is considered to be a sensitive reporter of the energy balance within the mitochondrium^[^ ^34^ ^]^ was found to continuously increase over the aging process. Further, we confirmed a mild, but significant increase of 5 complex I proteins in elderly individuals, among them two iron‐sulfur proteins, which play a critical role in transporting electrons through complexes I, II, and III to cytochrome c (see Table S4, Supporting Information “increased old annotated”).^[^ ^35^ ^]^ Thus, a disbalanced electron transfer may be one of the main sources of ROS generation during aging, leading to alterations in energy homeostasis.

Perturbances in the electron chain not only affect surrounding tissue but also alter the functionality of mitochondria themselves by causing ROS accumulation. ROS can disrupt the function of plasma membrane calcium ion pumps,^[^ ^36^ ^]^ which are present in the mitochondrial membrane. Additionally, it can increase the peroxidation of phospholipids located in the inner mitochondrial membrane, leading to depolarization, and allowing ions, such as calcium, to pass through.^[^ ^37^ ^]^ One mechanism of the mitochondria to counteract stressors is the opening of the mitochondrial permeability transition pore (PTP).^[^ ^38^ ^]^ PTP is a nonselective channel, allowing not only calcium ions but also other molecules such as cytochrome C or apoptosis and necrosis signaling molecules to transfer into the intermembrane space. It is assumed that ROS negatively affects the regulation of PTP, resulting in a constant opening of the pore, leading to a bioenergetic crisis and cell death. However, only one protein, PPIF, has been clearly identified to play a role in PTP opening by Ca^2^ or ROS challenge. In our study, we found that PPIF was increased twofold in elderly individuals compared to the young study group (see Table S4, Supporting Information “young versus old *t* test”). Additionally, we observed a significant increase in the abundance of its known interactor, SPG7^[^ ^39^ ^]^ in old aged individuals compared to the middle‐aged study group (see Table S4, Supporting Information “middle‐aged versus old *t* test”). Completing the picture, calpain‐1 and calpain‐2 were found to be enriched in elderly individuals compared to the young study group (see Table S4, Supporting Information “increased old annotated”). Calpains are cysteine proteases that are dependent on cytosolic calcium.^[^ ^40^ ^]^ Calpains are activated by an influx of calcium into the mitochondrial cytosol, making them particularly active under high calcium concentrations. Further, calpains interact with proteins involved in apoptosis and necrosis and are broadly associated with aging.^[^ ^41^ ^]^ Therefore, it is possible to speculate that changes in mitochondrial ROS generation may contribute significantly to the physiological aging of the SN.

A second factor contributing to ROS production in the SN is based on its unique composition being comprised, which is comprised of a high number of dopaminergic neurons. Monoamine oxidases catalyze the oxidative deamination of dopamine, leading to the generation of various ROS species.^[^ ^42^ ^]^ This is further increased by spontaneous dopamine auto‐oxidation in detail reviewed in.^[^ ^43^ ^]^ It is suspected, that SOD1 and glutathione peroxidases/transferases (GPX/GST) may play an important role in countering the neurotoxic effects of dopamine‐o‐quinones.^[^ ^44^ ^]^ Although SOD1 was not identified in our dataset, we found that its chaperone CCS was increased in the elderly, along with GSTT1, MGST3, GPX1, and GGT5. This suggests that ROS generation by dopamine metabolism may also contribute to the physiological aging of the SN.

In addition to the enzymes mentioned above, catalases, thioredoxins (TXNRD), and peroxiredoxins (PRDX) are also crucial in removing ROS species. This is aided by proteasome‐mediated protein degradation and autophagy. Our proteomic analysis revealed that two proteins associated with these functions were increased in elderly subjects, namely TXNRD1 and PRDX6 (see Table S4, Supporting Information “increased old annotated”). PRDX6 is already suspected to play a role in neurodegeneration since it was found to be highly expressed in reactive astrocytes of PD‐ and dementia with lewy body patients^[^ ^45^ ^]^ as well as astrocytes in close proximity to diffuse abeta plaques^[^ ^46^ ^]^ in Alzheimer's disease patients. A study utilizing an animal model of PD substantiated the hypothesis of PRDX6s pathophysiological function in dopaminergic neuron loss.^[^ ^47^ ^]^

Out of the 39 proteasomal proteins identified, six (PSMB5, PSMC1/2, PSMD1/3/4) were found to have decreased in elderly subjects (see Table S4, Supporting Information “young versus old *t*‐test”). This observation is consistent with recent literature, which suggests that proteasomes experience age‐related functional impairment in many tissues, including the brain. This impairment makes cells more reliant on alternative proteolytic pathways.^[^ ^48^, ^49^ ^]^ Interestingly, an inhibition of the ubiquitin‐proteasome pathway in PD was already hypothesized and verified, potentially triggering Lewy Body formation.^[^ ^50^ ^]^ A later study proposed a selective loss of the 20S proteasome alpha subunits in the SN of Parkinson's disease patients based on IHC staining and Western Blot.^[^ ^51^ ^]^ With our data we can substantiate the finding of a decreased abundance of proteasomal subunits, however, in our proteomic analysis subunits of the regulator complex and non‐ATPase subunits were found to be altered.

Finally, our study found a significant increase in proteins related to the complement cascade and immunomodulatory function, leading to neuroinflammation. In physiological aging mitochondrial dysfunction and alterations in the ubiquitin‐proteasome system, as present in our study, increase the chance of chronic inflammation.^[^ ^52^ ^]^ Confirmatively we identified 8 out of 31 acute phase proteins known to increase in plasma upon acute inflammation^[^ ^53^ ^]^ (see Table S4, Supporting Information “increased old annotated”). Among them ORM1, HP and CP display an FC > 4 and further two HLA class I antigens, whose primary function is to bind peptides generated from degradation of cytosolic proteins by the proteasome.^[^ ^54^ ^]^ These findings support our conclusion of an impaired proteasomal machinery.

An overproduction of inflammatory molecules can result not only from increased ROS but also from gliosis, such as astrogliosis. Under physiological conditions, astrocytes have numerous functions in the brain, including a structural role and supporting neurons in their energy demand. They also play a major role in CNS pathologies, as seen in astrogliosis, where they actively produce inflammatory molecules.^[^ ^55^, ^56^ ^]^ Although astrogliosis may have neuroprotective effects after acute damage, it is widely accepted that persistent activation, which occurs in the aging brain, leads to an overproduction of inflammatory molecules and an increased expression of GFAP.^[^ ^57^ ^]^ Immunohistochemical stainings of the human SN confirmatively verified an increasing expression of GFAP with age.^[^ ^21^ ^]^ This was also observed in our study. Astrocytes are the primary source of angiotensinogen (AGT) in the brain, which is the precursor peptide to angiotensin. This is a critical component of the renin‐angiotensin system, which is essential for brain homeostasis. The receptors of this system either promote or counteract vasoconstriction, proliferation, inflammation, and oxidative stress. Confirmatively we identified AGT to be upregulated in elderly subjects, alongside SERPINA3, which converts angiotensin‐1 to the active angiotensin‐2 (see Table S4, Supporting Information “increased old annotated”). An increase in inflammatory signals in the aging SN was further verified via immunohistochemistry by Beach et al.^[^ ^20^ ^]^ This adds another layer to the complex interplay between ROS generation, neuroinflammation, and the loss of structural integrity. These findings lead us to our next key aspect.

Elderly individuals display an increased number of proteins necessary for cytoskeletal structure, integrity, and remodeling. This may be connected to an altered vesicle traffic machinery, which can lead to difficulties in synaptic plasticity.

Throughout life, neurons in all brain regions are exposed to a variety of changes. Their ability to respond to these changes or stressors is called neuronal plasticity.^[^ ^58^ ^]^ At the cellular level, neuronal plasticity is reflected by changes to different stimuli in membrane excitability as well as in the anatomy of dendrites and axons.^[^ ^59^, ^60^ ^]^ An important factor hereby is the cytoskeleton. The adaptability of the cytoskeleton and thus the neuronal plasticity is mandatory to maintain, e.g., the morphology of the neuron or the transmission of signals along axons and by that its functionality.^[^ ^61^, ^62^ ^]^ Any changes, such as cellular stress, that occur during aging or a neurodegenerative disease in the cytoskeletal system can result in alterations in neuronal function. These alterations are manifested in changes to signaling pathways, potentially caused by a hindered vesicle trafficking machinery. Imbalances in the chemical reduction and oxidation of ROS were found to dysregulate actin and microtubule dynamics.^[^ ^63^, ^64^ ^]^ It is suspected that the redox balance regulates microtubules not only through direct interaction but also by regulating their stabilization through MAPs. Oxidation of MAP2 may lead to decreased microtubule polymerization in dendrites and the axon,^[^ ^53^ ^]^ potentially affecting vesicle trafficking, as observed in our analysis.

Our analysis revealed a significant increase in proteins essential for the structure, remodeling, and stability of the cytoskeleton in older individuals compared to our young study group. Specifically, proteins involved in the modulation of the actin network, including CAPN1, CAPN2, GSN, FLNA/B/C, MSN, and RDX, were found to be increased (see Table S4, Supporting Information “increased old annotated”).

However, proteins associated with organelles, such as essential regulatory elements for organelle positioning, e.g. SPAG9 and PIP4P1, organelle contact sites (VPS13A), and the integrity of the Golgi network (ARFGEF1/2/3) as well as the maintenance of mitochondrial morphology (OPA1), were found to be decreased in elderly individuals substantiating our findings in regards to mitochondrial alteration (see Table S4, Supporting Information “decreased old annotated”). Indeed, OPA1 deficiency is known to cause age‐related deficits in learning and memory, visible by the loss of neurons and a reduction in synaptic density.^[^ ^65^ ^]^ Further 3 proteins of the Arp2/3 complex were found to be decreased, which is controlling the nucleation of actin polymerization and branching of filaments and thus regulating the intracellular motility of lysosomes, endosomes, vesicles, and mitochondria. It is not unexpected that older people also showed reduced expression of 34 proteins essential for vesicle production, maturation, formation, and trafficking (see Table S4, Supporting Information “decreased old annotated”), suggesting changes in signaling pathways, neurotransmitter release, and synaptic plasticity in the aging brain.

Confirmatively, elderly individuals displayed a decreased abundance of proteins involved in neurite and axonal growth, dendrite morphogenesis, synaptic development, and neuronal plasticity. Therefore, it can be hypothesized that the aging brain attempts to compensate for the loss of structural integrity by increasing the production of proteins required for cytoskeletal remodeling and structure trafficking (see supplementary Table S4, Supporting Information “decreased old annotated”). This may be an effort to counteract an altered vesicle trafficking machinery that causes severe imbalances in neurotransmitter transport and release.

Aside from the active transport of vesicles, microtubules and actin filaments (in)directly transport molecules by the interaction between said molecules and membrane‐bound organelles attached to dynein/kinesin, also referred to as hitchhiking.^[^ ^66^ ^]^ Often hitchhikers are found to be ribonucleoprotein (RNP) granules, such as stress granules (SG). Irregularities in mRNA transport, as well as SG formation, may lead to detrimental effects, as local protein translation may be altered or disturbed, leaving the cell under‐cared for. Indeed, we identified several components of RNP and stress granules to be decreased in aged subjects. Among them ATXN2, and its interactor ATXN2L. Until recently, the function of ATXN2 was unknown, but its involvement in neurodegeneration through genetic variants leading to spinocerebellar ataxia type 2 and ALS, led to several discoveries highlighting its function in RNA processing, mRNA stability, and translation.^[^ ^67^, ^68^ ^]^ In addition, loss‐of‐function variants revealed their importance in the stress‐related induction of stress granules that protect vulnerable mRNA from decay.^[^ ^69^ ^]^ Further, STAU2 was found to be decreased in elderly individuals. Similarly, to ATXN2, it plays a crucial role in RNA translation by the regulation of neuronal target RNAs. Due to that, it has a pivotal role in neuronal synapses especially in regards to synaptic plasticity,^[^ ^70^ ^]^ adding another possible mechanism contributing to the observed decrease of proteins essential for synaptic transmission and plasticity in the elderly potentially leading to a degenerating brain (see Table S4, Supporting Information “increased old annotated”).

### 3.2 Proteins Associated with Neurodegenerative Diseases Display Alterations in Response to the Aging Process

As already explained physiological aging and neurodegeneration share several common mechanistics and the aging process is still defined as the major risk factor for developing a neurodegenerative disease. Thus, in our last chapter, we assessed abundance levels of proteins known to be risk factors of or altered in PD. For a comprehensive overview, we decided to create a table including information on the protein itself, its function in the cell, its possible interaction with other PD‐associated genes, and its abundance as identified in our study (see Table S4, Supporting Information “PD proteins and interactors”). The majority of proteins found to be differential in our analysis displayed decreased abundances in old individuals. At first glance, this may seem counterintuitive, but revisiting their function, it becomes evident, that their physiological function is connected to processes, that are proven to be altered in healthy physiological aging, e.g., SNCA, which is thought to play a role in synaptic vesicle mobility and recycling, as well as neurotransmitter release. Its interactor PRKN displayed a similar decrease.^[^ ^71^ ^]^ Under physiological conditions, PRKN is an essential mitochondrial E3 ubiquitin ligase, which promotes mitophagy and mitochondrial clearance maintaining mitochondrial quality.^[^ ^72^, ^73^ ^]^ Knock‐down of PRKN in murine model organisms led to mitochondrial impairment in the central nervous system, visible in an altered expression of mitochondrial proteins and a reduction in respiratory capacity.^[^ ^74^, ^75^ ^]^ A recent study showed that PRKN is inactivated by ROS, suggesting detrimental effects in conditions further escalating ROS production.^[^ ^76^ ^]^ Thus, we can speculate, that elevated ROS levels may lead to a reduced PRKN abundance, eventually resulting in an altered mitochondrial homeostasis. Besides SNCA, PRKN interacts with other PD‐related proteins, among them VPS35. Under physiological conditions, VPS35 localizes to endosomes and recruits proteins involved in autophagic processes.^[^ ^77^, ^78^, ^79^ ^]^ It is further involved in the maintenance of synapses, and dopamine transporter recycling and plays a key role in ensuring mitochondrial stability. VPS35 is known to interact with PRKN and PLA2G6 on the level of mitochondria, mediating the formation of mitochondria‐derived vesicles directed toward the peroxisome,^[^ ^80^ ^]^ stressing its essential function in mitochondria homeostasis and further substantiating the observed mitochondrial alteration in our study. In line with prior results, TMEM230, which shares a similar function as VPS35 was found to be decreased in the elderly. Under physiological conditions, TMEM230 plays an essential role in vesicle and retromer trafficking secretory autophagy and Golgi‐derived vesicle secretion.^[^ ^81^ ^]^ Expression of PD‐related mutations of TMEM230, led to a decreased speed of synaptic vesicle transport, contributing to a disturbed vesicle trafficking machinery in the elderly.^[^ ^82^ ^]^ Interestingly, studies in zebrafish models connect TMEM230 and angiogenic blood vessel growth, a pathway, of which several proteins were found to be altered in elderly individuals in our study as well, adding another layer of possible neurodegeneration‐contributing factors.^[^ ^83^ ^]^

In contrast to all other PD‐associated genes identified in our study, PARK7, was the only protein displaying an increased abundance during the aging process. Again, this observation may be based on its physiological function. PARK7 is a redox‐sensitive chaperone and a sensor of oxidative stress and can further inhibit SNCA aggregation. Increasing ROS levels in the SN caused by mitochondrial alterations and the dopamine metabolism may lead to an increased abundance of PARK7 to counteract against ROS‐induced damage, as demonstrated in several studies, whereby PARK7 deficiency leads to an increased ROS production, and overexpression to protection against ROS species.^[^ ^84^, ^85^, ^86^ ^]^

## 4 Conclusion

In this study, we were able to verify and substantiate known age‐related changes in the human brain on the level of the proteome, including mitochondrial abnormalities, increased ROS production as well as an increased inflammatory response, and connect these phenomena with the unique composition of the SN, and PD‐associated proteins. With our approach coupling laser microdissection and mass spectrometry we are able to detect age‐related changes in the proteome in a spatial manner, restricting analyses to the SN. Further, our approach enables the detection of proteomic changes utilizing minimal amounts of tissue, since only 2–8 10 µm tissue slides are needed for the complete workflow. However, working with a limited amount of tissues our study is not able to provide validation on hypotheses raised and may not be able to depict the proteome of the SN fully as certain protein classes, such as transcription factors, are too low abundant to identify when working with restricted amount of sample, which has to be considered when interpreting the data. Nevertheless, our study confirms well‐known age‐related alterations, such as an increase in ROS and inflammation highlighting the particular vulnerability of the SN and further connecting the role of known risk genes for PD by identifying alterations in associated pathways, mainly present in mitochondria, the ER and the Golgi apparatus.

## 5 Experimental Section

### Subjects

Post‐mortem brain tissue samples from 31 male and 4 female subjects aged 18–96 years were provided by the Newcastle Brain Tissue Resource UK, the Navarradbiomed Biobank of Pamplona and Biobank for Aging Studies at the University of São Paulo Medical School. The study group was divided into young subjects (*n* = 7 (all male), average age: 28.7 years), middle‐aged subjects (*n* = 14 (13 male, 1 female), average age: 62.3 years) and old‐aged subjects (*n* = 14 (11 male, 3 female), average age: 83.9 years)The subjects were healthy and died from natural causes, with no neurological or histopathological abnormalities detected. The use of human postmortem brain tissue was approved by the Ethics Committee of the Ruhr‐Universität Bochum (4760‐13), the Joint Ethics Committee of Newcastle and North Tyneside Health Authority, the Ethics Committee of Pamplona and the University of São Paulo, with written informed consent following brain bank procedures. Detailed information on the patient study group can be found in Table S1 (Supporting Information).

### Cryosectioning

Cryosectioning of frozen midbrains was performed by respective brain banks as described before.^[^ ^87^ ^]^ In short, at the level of the SN sectioning of frozen midbrains was carried out and 10 µm sections were placed on 1.0 PEN membrane glass slides (Carl Zeiss Microscopy GmbH, Göttingen, Deutschland) and stored at −80 °C until further usage.

### Staining and Laser Microdissection

To improve tissue visibility during the laser microdissection process, neuronal staining was performed using cresyl violet as previously described.^[^ ^87^ ^]^ Briefly, the slides were incubated for 2 min in 70% ethanol cooled at 4 °C, followed by staining with a 1% cresyl violet solution (1 g of cresyl violet acetate (Sigma Life Sciences, St. Louis, USA) dissolved in 50% ethanol) of 500 µL for 20 s. The slides were then washed in 70% and 100% ethanol cooled at 4 °C, respectively. Before dissection, the slides were air‐dried for 2 min. Laser microdissection was carried out following the protocol by Molina et al.^[^ ^6^ ^]^ Subsequently, the slides were placed on the PALM Micro Beam instrument (P.A.L.M.‐System LCM, Carl Zeiss Microscopy GmbH), and SN including stained neurons were marked at 400x magnification using the PALMRobo 4.6 Pro software (Carl Zeiss Microscopy GmbH, Göttingen, Germany). Cutting and catapulting were performed using the “RoboLPC” option with energy settings of 34–40 for cutting and 23–27 for LPC. The sample was transferred into the lid of a nonstick reaction tube (MicroTube 500, Carl Zeiss Microscopy GmbH, Göttingen, Germany) filled with 48 µL of ultrapure water (TKA‐Gene PURE, Thermo Scientific, Baltimore, USA). Approximately 2 000 000 µm^2^ of SN tissue, encompassing all cell types, was collected for each case. The reaction tubes were inverted and stored at −80 °C until required.

### Tryptic Digestion

Tryptic digestion was performed according to Molina et al.’s protocol with slight modifications.^[^ ^6^ ^]^ The digestion involved adding 7 µL of 1% RapidGest^TM^ SF Surfactant (Waters GmbH, Milford, MA, USA) resuspended in 50 mm ammonium bicarbonate. Then, cysteine residues were reduced and alkylated by adding 1,4‐dithiothreitol (final concentration 15 mm) for 30 min at 60 °C and iodoacetamide (final concentration 5 mm) for 30 min at room temperature in the dark. Tryptic digestion was performed at 37 °C for 4 h and stopped with 3.25 µL of 10% trifluoroacetic acid (TFA, Sigma Aldrich GmbH, Hofheim, Germany) at the same temperature for 30 min. Subsequently, RapidGestTM SF Surfactant was removed by centrifugation for 15 min at 4 °C and 10 000 rpm before MS measurements. Finally, the sample obtained after centrifugation was transferred to a glass vial and concentrated using a vacuum concentrator (Concentrator Plus, Eppendorf GmbH, Hamburg, Germany). The sample was reconstituted in 0.1% TFA to reach a final volume of 35 µL and stored at −80 °C for future analysis. Prior to mass spectrometry, an amino acid analysis was carried out in order to determine the peptide concentration of each sample as described before.^[^ ^88^ ^]^

### Mass Spectrometry

The LC‐MS/MS analysis was performed using an UltiMate 3000 RSLC nano‐LC system (Thermo Fisher Scientific, Bremen, Germany) coupled to a Q Exactive High Fusion system (Thermo Fisher Scientific, Bremen, Germany). Each sample (containing 350 ng peptides) was injected using an autosampler with 0.1% TFA at 60 °C and a flow rate of 30 µL min^−1^. After preconcentrating peptides for 7 min on a trap column (PepMap100, C18 phase, 100 µm ID × 2 cm, particle size 5 µm and pore size 100 Å), they were transferred to the analytical column (PepMap C18, C18 phase, 75 µm ID × 50 cm, particle size 2 µm, pore size 100 Å). The peptides were separated on an analytical column using a segmented gradient of 5–60% (v/v) running buffer B over 141 min. The solvent system consisted of running buffer A (0.1% formic acid) and running buffer B (84% acetonitrile, 0.1% formic acid). The HPLC system and Q Exactive High Fusion were online connected, and electrospray ionization was performed. Mass spectrometric measurements of the samples were carried out using data‐independent acquisition. The mass range for both MS1 and MS2 was set from 350 to 1100 m/z. Full scans were performed at a resolution of 120000 with an AGC target of 3e6, and a maximum injection time of 20 ms was set. Fragment ions were generated by HCD at NCE of 25.5%, 27%, and 30% with a fixed first mass of 130 m/z. There were 40 isolation windows with a width of 20 m/z and a window overlap of 1 m/z. The mass spectrometry proteomics data have been deposited to the ProteomeXchange Consortium^[^ ^89^ ^]^ via the PRIDE partner repository^[^ ^90^ ^]^ with the dataset identifier PXD051145.

### Data Analysis

The DIA data generated were evaluated using Spectronaut Pulsar software (version 16, Biognosys, Schlieren, Switzerland), with directDIA functionality, according to the manufacturer's default settings with minor adjustments: The produced data files are available in the public repository PRIDE with the identifier PXD051145. Trypsin was specified as the digestion enzyme, carbamidomethylation (C) was fixed, and methionine oxidation was dynamic. The original Spectronaut analysis output can be found in Table S2 (Supporting Information). Normalization was disabled and carried out using an in‐house R‐script that utilized LOESS normalization (see Table S3, Supporting Information). Normalized data was transformed logarithmically, and missing values were imputed from a normal distribution with a width of 0.3 and a downshift of 1.8. To identify proteins gradually increasing or decreasing with age, one‐way ANOVA (*p*‐value < 0.05) and subsequent PostHoc Tukey's HSD test for one‐way ANOVA (FDR 0.05) to determine significant pairs between the three groups were utilized. Additionally, to identify differential proteins between two groups, an unpaired student's *t*‐test was performed, and proteins with a *p*‐value < 0,05 were assigned to be significantly expressed. Fold changes (FC) were calculated by subtracting the log2 label‐free quantified (LFQ) values.

## Conflict of Interest

The authors declare no conflict of interest.

## Author Contributions

K.M. and C.M. performed conceptualization and project administration. I.G.A, S.K., M.M., L.T.G., H.H., R.E.P., J.A. performed Collection and preparation of brain specimens. S.S. performed staining laser microdissection and tryptic digestion. S.S., B.E performed mass spectrometric measurements. B.E., S.S., and C.M. performed data analysis. B.E. I.G.A., S.K, M.M., L.T.G., H.H., R.E.P., J.A, C.M. performed data curation. B.E. C.M. performed the creation of tables and figures. B.E., S.S. Wrote the original draft. All authors reviewed the manuscript.

## Supporting information



Supporting Information

Supplemental Table 1

Supplemental Table 2

Supplemental Table 3

Supplemental Table 4

## Data Availability

The data that support the findings of this study are openly available in ProteomeXChange at https://proteomecentral.proteomexchange.org/ui, reference number 51145.
