# Supplementary material for: The Aging Substantia Nigra is Characterized by ROS Accumulation Potentially Resulting in Increased Neuroinflammation and Cytoskeletal Remodeling
Source: Adv Biol (Weinh). 2025 Mar 12;9(4):2400814. doi: 10.1002/adbi.202400814 (PMC12001008; doi:10.1002/adbi.202400814)
Supplement: Supplementary file 1 — Supporting Information [file ADBI-9-2400814-s002.pdf]

ADVANCED BIOLOGY

Supporting Information

for *Adv. Biology*, DOI 10.1002/adbi.202400814

The Aging Substantia Nigra is Characterized by ROS Accumulation Potentially Resulting in Increased Neuroinflammation and Cytoskeletal Remodeling

Britta Eggers, Simone Steinbach, Isabel Gil Aldea, Sharon Keers, Mariana Molina, Lea T. Grinberg, Helmut Heinsen, Renata E. Paraizo Leite, Johannes Attems, Caroline May and Katrin Marcus**

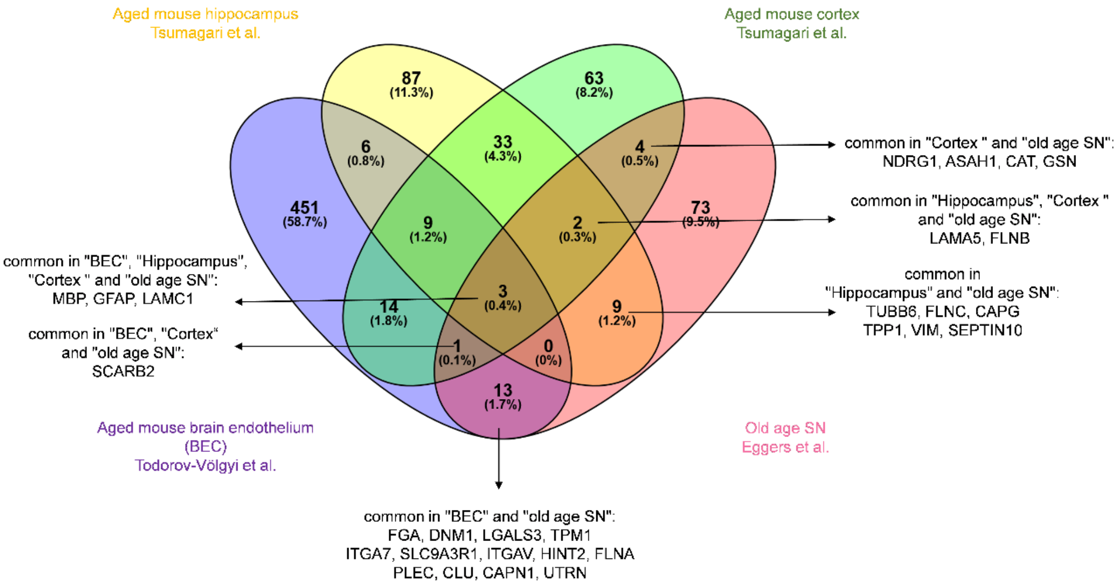

Supplementary figure S1: Venn Diagram (Oliveros, J.C. (2007-2015) Venny. An interactive tool for comparing lists with Venn's diagrams. https://bioinfogp.cnb.csic.es/tools/venny/index.html) comparing proteins identified being of higher abundance in aged mouse hippocampus, aged mouse cortex, aged mouse brain endothelium and aged human substantia nigra samples. Enrichment of proteins was always verified by statistical comparison to a younger cohort of the same sample type. Proteomic data was taken from Tsumagari et al. and Todorov Völgyi et al. Proteins overlapping between the different data sets are indicated by an arrow. For interpretation purposes the gene name is added.
